# Anxiety and Depressed Mood in Older Patients Peri-Hospitalization: The Role of Physical Symptoms and Sleep quality

**DOI:** 10.1093/geroni/igaf122.2699

**Published:** 2025-12-31

**Authors:** Juliana Smichenko, Anna Zisberg, Tamar Shochat

**Affiliations:** University of Haifa, Haifa, HaZafon, Israel; University of Haifa, Haifa, HaZafon, Israel; University of Haifa, Haifa, HaZafon, Israel

## Abstract

Anxiety and depression are common among older adults affecting independence and well-being. However, their trajectories during the peri-hospital period remain unclear. This study examined changes in anxiety and depression from admission to discharge and one-month post-discharge, focusing on the roles of physical symptoms burden and sleep quality, and controlling for sedative-hypnotic medications burden. Data were analysed from the Hospitalization Process Effects on Mobilization Outcomes and Recovery (HoPE-MOR) study, including adults aged 65+ hospitalized in acute medical units. Assessments occurred at admission, discharge, and one-month post-discharge. A two-stage modelling approach examined mood changes from admission to discharge in 683 patients; and (out of them) from admission to post-discharge in 545 patients, controlling for demographic and baseline characteristics. Patients were aged 77.31±6.67, with 55% males. Results indicated worse anxiety at post-discharge compared to admission and discharge, and a decline in mood from admission through post-discharge. Poorer sleep quality, increased physical symptom burden (B(exp)anx=.38,p <.001;B(exp)dep=.043,p=.02), and sedative-hypnotic medication (SHM) burden (B(exp)anx=0.512,p <.001) significantly predicted anxiety and depression during and after hospitalization. Sleep quality effects varied by timepoint: poorer sleep was linked to increased anxiety post-discharge (F(2,879)=7.52,p <.001) and greater depressive symptoms during hospitalization (F(2,874)=7.99,p <.001). Additionally, higher SHM burden was associated with worsened depressive mood at discharge (F(2,874)=4.80, p<.01). Findings underscore the importance of addressing anxiety and depression during hospitalization, emphasizing the need to improve sleep quality, manage physical symptoms, and regulate SHM use. Future research should explore longer follow-up periods and modifiable factors to enhance mental well-being throughout hospitalization and recovery.

